# Analysis of the therapeutic effect and prognosis in 86 cases of rib fractures and atelectasis

**DOI:** 10.1186/s13018-021-02221-y

**Published:** 2021-01-28

**Authors:** Degang Yin, Jingang Lu, Jiansheng Wang, Biao Yan, Zhongshu Zheng

**Affiliations:** 1 Department of Thoracic Surgery, Xixi Hospital, Hengbu Street 2, Xihu District, Hangzhou, 310023 China; 2 Department of Anesthesiology, Xixi Hospital, Hangzhou, 310023 China; 3 Second Department of Surgery, Xixi Hospital, Hangzhou, 310023 China

**Keywords:** Thoracic trauma, Rib fracture, Atelectasis, Pulmonary reexpansion

## Abstract

**Background:**

The aim of the present study was to explore the therapeutic effect and prognosis in patients with rib fractures and atelectasis after thoracic trauma in order to provide a basis for clinical decision-making in primary hospitals.

**Methods:**

A retrospective study was conducted on 86 patients admitted to our hospital between January 2016 and May 2020 with rib fractures and atelectasis after thoracic trauma. On the basis of the chest computed tomography scans taken at the time of discharge, the patients were divided into two groups: the reexpansion group and the non-reexpansion group. The two groups were compared with respect to the changes observed in the patients’ levels of blood oxygen saturation (SpO2) and pulmonary function, the presence of secondary pulmonary or thoracic infection, the time of chest tube drainage, the length of hospitalization, the cost of hospitalization, and the patients’ level of satisfaction with their quality of life 3 months after discharge.

**Results:**

In the reexpansion group, there were significant differences in the levels of SpO2 and pulmonary function measured before and after pulmonary reexpansion (*P* < 0.05). Compared with the non-reexpansion group, the patients in the reexpansion group had a lower incidence of secondary pulmonary and thoracic infection and a higher level of satisfaction with their quality of life after discharge; these differences were statistically significant (*P* < 0.05). There was no statistical significance between the two groups with respect to the time of chest tube drainage or the length of hospitalization (*P* > 0.05). However, the cost of hospitalization was significantly higher in the reexpansion group than in the non-reexpansion group (*P* < 0.05).

**Conclusions:**

The patients in the pulmonary reexpansion group had a lower incidence of complications and a better prognosis than the patients in the non-reexpansion group.

## Background

The incidence of thoracic injuries has gradually increased as injuries caused by traffic accidents become more common. Atelectasis is associated with bronchial stenosis or obstruction, a decrease in the volume of gas in the lung, and a reduction in pulmonary volume. Although atelectasis can have many different causes, it is most commonly caused by thoracic trauma, especially in patients with rib fractures. Atelectasis caused by thoracic trauma can be accompanied by many other conditions. The delayed or improper treatment of atelectasis can seriously affect the quality of life and prognosis of patients and lead to pulmonary infection, hypoxemia, and even death.

## Methods

### Materials

From January 2016 to May 2020, 1351 patients with rib fractures were treated in our hospital. After patients whose rib fractures were caused by non-traumatic factors or whose fractures were not being treated for the first time were excluded, there were 1102 patients (783 males and 319 females) aged 21–88 years. The reasons for the patients’ rib fractures were as follows: traffic accidents (756 cases), falls (110 cases), falls from height (188 cases), sharp instruments (8 cases), and blunt instruments (40 cases). The types of trauma were as follows: closed injury (1093 cases) and open injury (9 cases). The number of rib fractures ranged from 1 to 16 and affected the first rib to the twelfth rib. There were 30 cases of flail chest, 667 cases of hemothorax, 140 cases of pneumothorax, 405 cases of lung contusion and laceration, and 712 cases that were complicated by injuries to other sites. The cases with injuries at other sites included 86 cases of atelectasis with an incidence of 7.8%. There were 62 cases of partial lobular atelectasis, 24 cases of lobular atelectasis, and no cases of total pulmonary atelectasis. There were 56 cases of atelectasis-related complications, including 53 cases of pulmonary infection and 3 cases of acute lung injury and acute respiratory distress syndrome (ARDS). No patients died. All cases of atelectasis were confirmed by chest computed tomography (CT) examination during the period of hospitalization after the injury. After treatment, the chest CT results at the time of discharge were used to divide the 86 patients with atelectasis into two groups: the reexpansion group (49 cases) and the non-reexpansion group (37 cases). As shown in Table 1, there were no significant differences in the general characteristics of the two groups (all *P* > 0.05).

**Table 1:** Comparison of the general characteristics between the two groups

Group	*n*	Age (years)	Thoracic AIS score (points)	Number of fracture (*n*)	Pulmonary contusion (*n*)	Hemothorax (*n*)	Pneumothorax (*n*)	Flail chest (*n*)
Reexpansion group	49	47.33 ± 7.56	4.16 ± 0.99	5.16 ± 2.02	49	49	39	16
Non-reexpansion group	37	46.15 ± 6.64	4.03 ± 0.92	4.99 ± 1.88	37	37	35	14

### Methods

The patients were treated with thoracic external fixation, oxygen inhalation, and hemostatic, analgesic, anti-infection, and anti-shock therapies, as well as other conventional treatments after hospitalization. Depending on the amount of gas in the pneumothorax and the pleural effusion, pleural puncture and chest tube drainage were used. If necessary, urokinase was injected into the pleural cavity to dissolve blood clots. In the event of dyspnea or respiratory distress, positive pressure ventilation was used or sputum aspiration and lavage performed via bronchofiberscope. If reexpansion could not be achieved with any of these treatments, surgical treatments to promote pulmonary reexpansion were potentially used, including rib reduction, internal fixation, chest exploration, fiberboard stripping, and lung expansion during the operation.

**Fig. 1:**
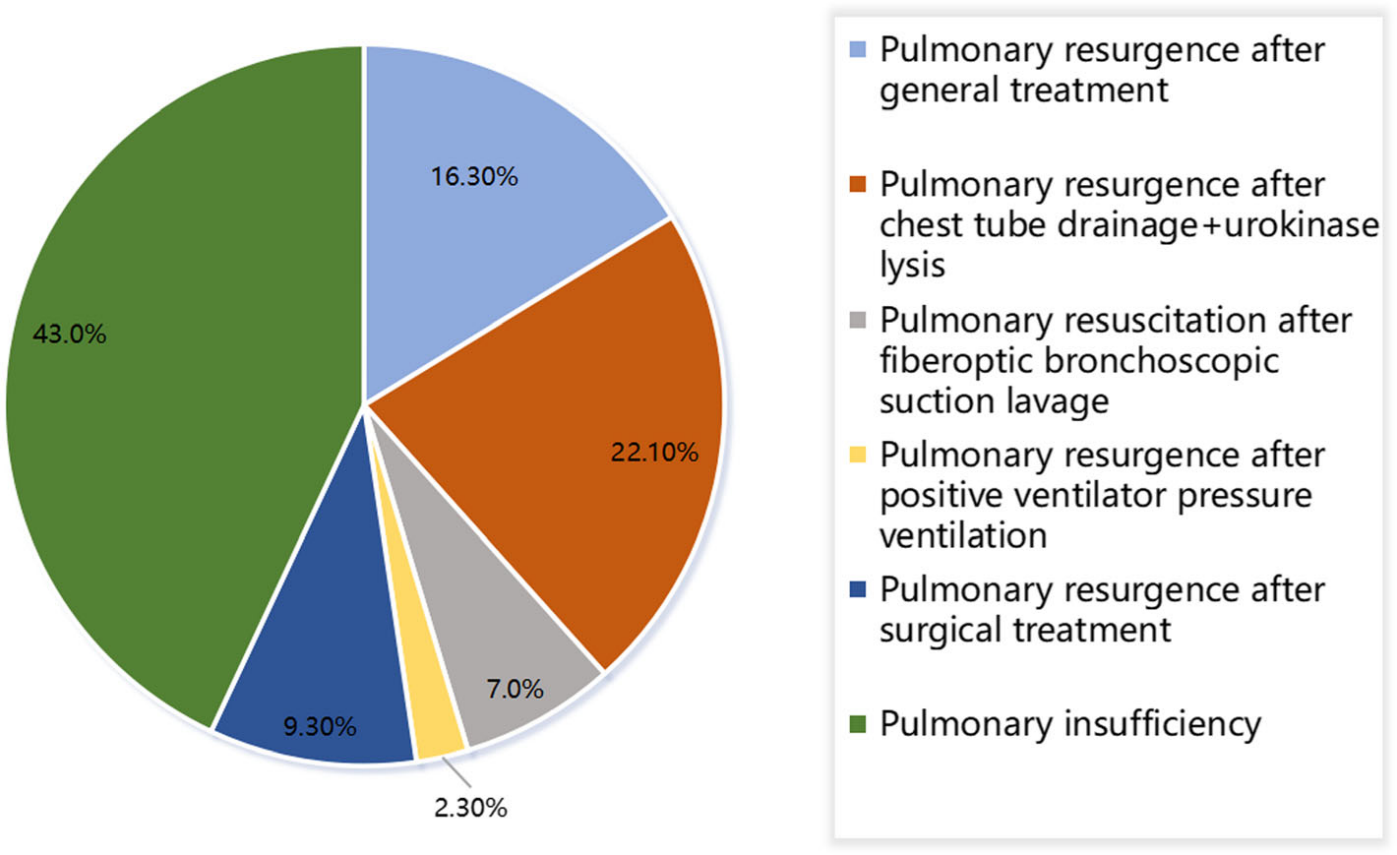
The therapeutic outcome in 86 patients with traumatic rib fracture accompanied by atelectasis

In 14 cases (16.3%), pulmonary reexpansion was achieved by conventional treatments. In 9 cases (22.1%), reexpansion was achieved by thoracic drainage, where 8 of these cases required thrombolysis via the injection of urokinase into the thoracic drainage tube. In 6 cases (7%), reexpansion was achieved by sputum aspiration and lavage via bronchofiberscope; in 2 of these cases, aspiration and lavage were combined with rib internal fixation. In 2 cases (2.3%), reexpansion was achieved by positive pressure ventilation. In 8 cases (9.3%), reexpansion was achieved after the elements that were restricting lung expansion were surgically removed. In the remaining 37 cases (43%), reexpansion was not achieved. These outcomes are shown in Fig. 1.

### Observation indices

The two groups were compared before and after treatment with respect to the changes observed in the patients’ levels of blood oxygen saturation (SpO2) and pulmonary function. Chest CT scans were performed and examined regularly. Sputum and pleural effusion cultures were used to determine drug sensitivity. The time of chest tube drainage, length of hospitalization, and cost of hospitalization were statistically analyzed. The two groups were also compared in terms of the patients’ level of satisfaction with their quality of life 3 months after discharge.

### Criteria for surgical treatment

1. Pulmonary atelectasis that fails to reopen despite multiple conservative treatment measures
2. An encapsulated pleural effusion that cannot be effectively drained
3. Where a fibrous membrane has formed on the surface of the lung
4. Coagulated hemothorax, or chronic abscess chest, which has formed a fibrous plate and requires surgical debridement
5. Multiple rib fractures, thoracic collapse, and flail chest, who require surgical treatment with internal rib fixation and may undergo pulmonary resuscitation surgery at the same time
6. Those who have solid fibrosis of the pulmonary tissues of the atelectasis, requiring surgical resection

### Statistical analysis

The SPSS 20.0 software (IBM Corp., Armonk, NY, USA) was used for data analysis. The measurement data with a normal distribution were expressed as mean ± standard deviation (x̄ ± SD), and the comparison between groups was conducted via Student’s *t* test. The enumeration data were analyzed with the *χ*^2^ test. *P* < 0.05 was considered statistically significant.

## Results

The results are summarized in Table 2. In the reexpansion group, there were statistically significant differences in the levels of SpO2 and pulmonary function measured before and after pulmonary reexpansion (*P* < 0.05). In addition, the patients in the reexpansion group had a lower incidence of secondary pulmonary and thoracic infection and a higher satisfaction with their quality of life after discharge than the patients in the non-reexpansion group did; both differences were statistically significant (*P* < 0.05). There were no statistically significant differences in the two groups’ time of chest tube drainage or length of hospitalization (*P* > 0.05). However, the cost of hospitalization was significantly higher for the reexpansion group than it was for the non-reexpansion group (*P* < 0.05).

**Table 2:** Comparison of the therapeutic effect and prognosis between the two groups

Group	*n*	SpO2 (%)		Changes in the pulmonary function FEV1%		Secondary pulmonary infection (*n*)	Secondary thoracic cavity infection (*n*)	Chest tube drainage time (days)	Length of stay (days)	Expense of hospitalization (Yuan)	Quality of life satisfaction (%)
		Before reexpansion	After reexpansion	Before reexpansion	After reexpansion						
Reexpansion group	49	88.90 ± 1.1	98.2 ± 0.17*	60.80 ± 1.36	82.13 ± 0.15*	13*	5*	8.02 ± 1.5	20.98 ± 4.5	29286.52 ± 8321.89*	96*
Non-reexpansion group	37	90.10 ± 1.06		61.00 ± 1.03		26	15	7.88 ± 3.3	19.67 ± 5.9	8686.56 ± 2102.6	56.25

Compared with the group with conservative therapy, ^*^P < 0.05

## Discussion

Atelectasis, a common complication of thoracic trauma, involves a decrease in the amount of air in certain bronchial and pulmonary segments and results in the atrophy of pulmonary tissue. Atelectasis may occur in any part of the lung and may be accompanied by the consolidation of the alveolar cavity.

### The causes and possible mechanisms of atelectasis after thoracic trauma

There are many potential causes of atelectasis after thoracic trauma, including obstruction, compression, fiber wrapping, and reduced levels of alveolar surfactant. Of these, atelectasis is most commonly caused by obstruction, compression, or a combination of factors. The major causes of atelectasis are summarized below.

1. Thoracic trauma can directly result in pulmonary hemorrhage and edema, which reduce alveolar compliance and ventilation and levels of alveolar surfactant. These changes can eventually cause alveolar collapse, resulting in atelectasis.
2. Thoracic trauma is often accompanied by bronchial–pulmonary injury. When bronchioles are broken and injured, blood can enter, and blood or phlegm clots can block the distal bronchioles, leading to atelectasis. Bronchial obstruction is the most common cause of atelectasis, which occurs when gas in the distal bronchioles is gradually absorbed, resulting in alveolar collapse and atelectasis.
3. After thoracic trauma, the bony structure of the thorax is damaged. Hemopneumatosis in the thorax can compress the alveoli, leading to compressive atelectasis. Secondary pulmonary infection can also encourage the development of atelectasis, which most often affects the lower lobe of the infected lung.
4. Chest pain caused by thoracic trauma may lead to respiratory restriction, shortness of breath, and incomplete expansion of the alveoli. As a result of the pain, patients avoid coughing, which leads to the accumulation of sputum that is difficult to cough up and that can block the bronchus.
5. Thoracic trauma that is accompanied by a disturbance of consciousness is more likely to cause aspiration and obstruction of the airway, resulting in atelectasis.
6. When body position is restricted after thoracic trauma, blood gas distribution is affected, which can cause the lower part of the pulmonary tissue to collapse.
7. Excessive fluid infusion can also lead to pulmonary reperfusion injury, resulting in pulmonary edema and atelectasis.
8. Thoracic trauma is often accompanied by rib fracture, pulmonary injury, and hemopneumothorax. Pulmonary contusion is an important factor contributing to atelectasis. In patients with pulmonary contusion, cellulose is deposited on the pulmonary surface, reducing pulmonary compliance and leading to atelectasis. Some researchers have suggested that simple pulmonary contusion can cause atelectasis. In cases of multiple rib fractures in which the thoracic wall has collapsed, abnormal breathing can also lead to atelectasis.

The factors discussed above promote the occurrence and development of atelectasis after thoracic trauma, both on their own and in combination, and atelectasis can lead to more serious complications, such as hypoxemia. In the present study, 31 cases of atelectasis were caused by pulmonary contusion. Of these, 28 cases were complicated by compression due to massive hemothorax, and 5 were caused by airway obstruction. The patients exhibited different degrees of rib fracture, which suggests that more attention should be paid to cases of atelectasis in which rib fractures are complicated by pulmonary contusion and a degree of pleural effusion.

### The treatment of thoracic trauma combined with atelectasis

The therapeutic goals for the treatment of atelectasis are the elimination of the factors that caused it, the promotion of pulmonary reexpansion, the restoration of pulmonary function, and the correction of hypoxemia in time. The measures used to treat atelectasis are summarized below.

Conventional therapy for atelectasis consists of several measures. First, oxygen inhalation, external thoracic fixation, and symptomatic analgesic treatment were used in addition to keep the patient’s airway unobstructed. Oxygen inhalation should be used to dilute the sputum and, in order to avoid pulmonary edema, the amount of crustal fluid infusion should be controlled. Second, antibiotics are administered rationally. Thoracic trauma with atelectasis is often accompanied by a degree of pulmonary contusion, and a long period of bed rest can lead to hypostatic pneumonia. Therefore, it is necessary to use antibiotics in a rational manner, making adjustments in response to the drug sensitivity of the sputum culture and the patient’s experience. Third, postural drainage is performed. For patients that can turn over, postural drainage should be performed, and patients should be taught the technique of patting. In the present study, 14 patients achieved pulmonary reexpansion through the use of conventional therapy, 11 patients required automatic discharge or hospital transfer, and all other patients required treatment with other therapies.

For patients with more than a moderate amount of pleural effusion or a degree of pulmonary compression greater than 30%, closed thoracic drainage should be performed. Coagulative hemothorax is the main cause of compressive atelectasis. After a massive hemorrhage in the thoracic cavity, the effect of defibrin is reduced, and a large number of blood clots form in the thoracic cavity, compressing the pulmonary tissue and resulting in atelectasis. In clinical settings, urokinase is often used to counter these effects. When urokinase is injected into the thoracic cavity, it disintegrates blood clots and improves drainage. In addition, it can dissolve the cellulose deposited on the pulmonary surface, which makes pulmonary reexpansion possible. Because this method is simple and provides a way of treating patients without great economic burden or special equipment, it deserves broader application [1]. In the present study, 61 patients were eligible for thoracic puncture and chest tube drainage. The procedure was performed on 50 patients, while 11 other patients were discharged after refusing intubation. Of the 19 patients that achieved pulmonary reexpansion after intubation, 8 cases first required blood clots to be dissolved by the intrapleural injection of urokinase; the patients that did not achieve reexpansion after intubation were subsequently treated with other methods.

In patients with hypoxemia caused by severe rib fractures and atelectasis, ventilator-assisted breathing should be performed. If necessary, positive end-expiratory pressure therapy should be used to reduce the dead space of the respiratory tract, increase pulmonary oxygenation, promote pulmonary reexpansion, reduce capillary fluid leakage, and improve pulmonary edema. In the present study, 3 patients with ARDS were treated with positive pressure ventilation, and 2 achieved pulmonary reexpansion; the patient that did not achieve reexpansion was subsequently treated with other methods.

If atelectasis persists after the therapies discussed above are used, further examination and treatment can be performed using fiberoptic bronchoscopy. Studies have shown that bronchoalveolar lavage (BAL) plays an important role in the diagnosis and treatment of atelectasis. A fiberoptic bronchoscope can be placed in a bronchus under direct vision, where it can reach the lesion and explore the condition of the airway. On the basis of the exploratory investigation, the causes of the obstruction can be analyzed and the lavage scheme formulated.

### The necessity of surgical treatment

To release the encapsulating separation of fluid in the chest cavity to facilitate drainage of the pleural fluid; to remove the fibrous membrane from the surface of the affected lung or the intra-thoracic fibrous plate to restore thoracic support and remove extra-pulmonary restrictive factors; if the atelectasis gradually evolves into a solid lung, etc., surgical resection is required to reduce the dead space.

BAL has a number of advantages. First, it can be used to quickly and accurately remove the thrombus and mucus secretion in the airway and bronchi under direct vision. Second, if the sputum block and purulent secretion cannot be directly aspirated, then BAL can be used to apply normal saline or antibiotic and hormone solution to wash the airway repeatedly, relieving the airway obstruction and alleviating inflammation [2–7]. Because the alveolar wall is thin and has a rich capillary network, solutions that are administered locally will be absorbed more easily [8], which will accelerate the patient’s improvement. Third, fiberoptic bronchoscopy can be used to determine the etiology of atelectasis by means of a biopsy, or exfoliated cells can be collected for cytological examination and culture [9–11]. Fourth, fiberoptic bronchoscopy has been successfully used to treat atelectasis by locally expanding the respiratory sac [12]. In addition, mechanical ventilation can be using alongside BAL to keep the patient’s respiratory tract under continuous positive pressure and achieve pulmonary reexpansion [13].

Fiberoptic bronchoscopy and BAL can be used repeatedly for pulmonary reexpansion, and these therapies have both a precise effect and a potentially broad application. In the present study, 9 patients were treated with fiberoptic bronchoscopy, 6 of whom achieved reexpansion. Of those 6 patients, 2 patients had thoracic trauma, and their fractured ribs were successfully treated with open reduction and internal fixation. The other 3 patients initially had a poor effect after sputum suction but ultimately achieved reexpansion after the operation. The patients that did not achieve reexpansion were subsequently treated with other methods.

Patients with multiple rib fractures can be treated with open reduction and internal fixation. Once the effusion and blood clot have been removed, bronchoscopy can be used for sputum suction during open reduction and internal fixation, and the effect of pulmonary reexpansion can be observed under direct vision. A large number of clinical studies have demonstrated that, in cases of multiple rib fractures, treatment with internal fixation is superior to the conservative treatments used to prevent pulmonary complications like atelectasis [14–19].

Open reduction and internal fixation was indicated for patients with the following characteristics: (1) patients with thoracic trauma accompanied by rib fractures, visceral injuries, or hemopneumothorax requiring thoracotomy; rib reduction and internal fixation could be performed at the same time; (2) patients with thoracic collapse and abnormal breathing, flail chest with dyspnea, or an obvious thoracic deformity, where surgery is required to restore the shape of the thoracic cavity; and (3) patients with any of the following conditions: (a) multiple rib fractures (multiple sites on multiple ribs) with more than three multiple rib fractures (multiple sites on multiple ribs) with dislocation of the fracture ends; (b) five or more single rib fractures with dislocation of the fracture ends and intractable chest pain; (c) a fracture site with special properties, with serious dislocations that pose a risk to vital blood vessels and organs; (d) fracture ends that were dislocated and failed to heal after conservative treatment for at least 2 months; (e) higher requirements for quality of life; and (f) no serious osteoporosis or systemic complications, with no osteomyelitis or pyogenic infection in the local area.

Previous studies have demonstrated that patients that underwent open reduction and internal fixation experienced a significant reduction of chest pain and an effective recovery of cough, which aids expectoration [20–22]. For these patients, getting out of bed early in the postoperative period not only promoted recovery but reduced the incidence of atelectasis.

If patients develop an encapsulated pleural effusion or fiberboard, it is often necessary to clean the pleural cavity and peel off the fiberboard. During the operation, the anesthesiologist should repeatedly aspirate the sputum to drum up the lung and promote pulmonary reexpansion. If the patient has a tracheal injury, active surgical repair will also be necessary [23].

In the present study, 8 patients achieved pulmonary reexpansion through the surgical removal of pulmonary restrictions, while 15 cases were discharged after refusing surgical treatment. Due to the high costs associated with surgery and the use of ventilators, the cost of hospitalization of the reexpansion group was significantly higher than that of the non-reexpansion group.

During the course of our study, we found that a considerable proportion of patients (43%) lacked an understanding of atelectasis, or did not find it worthy of attention. Some patients even had a fluke mentality and hoped to achieve recovery through conservative treatments alone. Because no patients died during the course of the study or the follow-up period, we were able to evaluate the patients’ prognoses by asking about their satisfaction with their quality of life after discharge. We found that some patients in the non-reexpansion group gradually achieved reexpansion within 3 months of discharge (22%); this may have been the result of the patients gradually absorbing the pleural effusion and having had a mild degree of atelectasis. These patients also reported a higher quality of life than were reported by patients that failed to achieve reexpansion. However, the reexpansion group reported a higher quality of life than the non-reexpansion group did, and this difference was statistically significant. This indicates that the reexpansion group had a better prognosis than the non-reexpansion group. Therefore, we recommend that atelectasis be addressed and treated as proactively and correctly as possible in order to achieve pulmonary reexpansion and improve the patient’s prognosis and quality of life.

For conservative treatment measures, our recommendations are as follows:

1. Prompt drainage and maintenance of unobstructed drainage, with improved drainage if necessary, to relieve extra-pulmonary compression restrictions
2. The need for positive pressure ventilation ventilator assisted therapy (PEEP) in some critically ill patients, which can reduce airway dead space and improve pulmonary oxygenation, while reducing capillary fluid leakage and improving pulmonary edema
3. Timely review of chest CT for lung condition and promptly aspirate and lavage with fiberoptic bronchoscopy as often as necessary, both to relieve intrapulmonary obstructive factors and to facilitate inflammatory regression. For patients for whom surgical treatment is indicated, we recommend surgery to treatment

## Conclusions

Atelectasis is a common complication of thoracic trauma that may lead to secondary complications if it is not treated promptly and correctly. When effective comprehensive treatments are used in a timely manner, patients with atelectasis can experience an improved therapeutic effect and have a better prognosis.

## Data Availability

All data generated or analyzed during this study are included in this published article.

## Abbreviations

ARDS: Acute respiratory distress syndrome
CT: Computed tomography
BAL: Bronchoalveolar lavage

## References

[1] Huang D, Zhao D, Zhou Y (2016). Intrapleural fibrinolytic therapy for residual coagulated hemothorax after lung surgery[J]. World J Surg.

[2] Li F, Zhu B, Xie G, Wang Y, Geng J. Effects of bronchoalveolar lavage on pediatric refractory mycoplasma pneumoniae pneumonia complicated with atelectasis: a prospective case-control study [published online ahead of print, 2020 Apr 2]. Minerva Pediatr. 2020. 10.23736/S0026-4946.20.05538-3. 10.23736/S2724-5276.20.05538-3 32241100

[3] Min Y, Dehua Y, Xin Y, Yingshuo W, Lei W, Min CZ (2018). Efficacy of bronchoalveolar lavage and its influence factors in the treatment of *Mycoplasma pneumoniae* pneumonia with atelectasis. Chinese J Pediatrics.

[4] Hongsheng D (2015). 20 cases of obstructive pulmonary insufficiency due to multiple rib fractures treated by bedside fiberoptic bronchoscopy. Chin J Rural Med Pharmacy.

[5] Yanhui S (2016). Results of bedside fiberoptic bronchoscopy for obstructive pulmonary insufficiency due to multiple rib fractures[J].China practical. Medicine.

[6] Dejun L (2018). Observation of clinical outcomes of electronic bronchoscopic alveolar lavage for refractory pulmonary insufficiency in children[J]. China Med Device Inform.

[7] Faro A, Wood RE, Schechter MS (2015). Official American Thoracic Society technical standards: flexible airway endoscopy in children[J]. Am J Respir Crit Care Med.

[8] Yingwei R (2019). Analysis of the effect of fiberoptic bronchoscopy combined with aminobronchol lavage on the NIHSS score, CRP, PCT, and WBC of patients with cerebral infarction with severe pulmonary infection with pulmonary insufficiency. Chin Foreign Med Res.

[9] Yang M, Yang DH, Yang X (2018). Efficacy of bronchoalveolar lavage and its influence factors in the treatment of *Mycoplasma pneumoniae* pneumonia with atelectasis[J]. Zhonghua Er Ke Za Zhi.

[10] Liu J, Ren XL, Fu W (2017). Bronchoalveolar lavage for the treatment of neonatal pulmonary atelectasis under lung ultrasound monitoring. J Matern Fetal Neonatal Med.

[11] Bernhard C, Masseau I, Dodam J (2017). Effects of positive end-expiratory pressure and 30% inspired oxygen on pulmonary mechanics and atelectasis in cats undergoing non-bronchoscopic bronchoalveolar lavage. J Feline Med Surg.

[12] Lei C, Jianguo G, Yong X, Dongying Z, Huiyan C, Ying Z (2015). The treatment of high pressure inflation through bronchoscope recruit local pulmonary atelectasis caused by segment injury[J]. China Health Standard Manage.

[13] Zhifeng H, Mingxia L, Jieyong C, Fengxia L (2016). Efficacy of bronchoalveolar lavage and positive pressure ventilation in treatment of traumatic atelectasis. Chin J Biomed Eng.

[14] Marasco S, Quayle M, Summerhayes R (2016). An assessment of outcomes with intramedullary fixation of fractured ribs. J Cardiothorac Surg.

[15] Pieracci FM, Leasia K, Whitbeck S (2019). Barriers to conducting a multi-center randomized controlled trial of surgical stabilization of rib fractures (and how to overcome them). J Thorac Dis.

[16] Wijffels MME, Prins JTH, Polinder S (2019). Early fixation versus conservative therapy of multiple, simple rib fractures (FixCon): protocol for a multicenter randomized controlled trial. World J Emerg Surg.

[17] Zhang JP, Sun L, Li WQ (2019). Surgical treatment of patients with severe non-flail chest rib fractures.[J]. World J Clin Cases.

[18] Xia H, Zhu D, Li J (2020). Current status and research progress of minimally invasive surgery for flail chest.[J]. Exp Ther Med.

[19] Yunping L, Xianguo C, Yiming N (2012). Review of 96 cases of treatment of pulmonary insufficiency due to chest trauma. Zhejiang Trauma Surg.

[20] Wang HC, How CH, Lin HF (2017). Traumatic left main bronchial rupture: delayed but successful outcome of robotic-assisted reconstruction[J]. Respirol Case Rep.

[21] Berland M, Oger M, Cauchois E (2018). Pulmonary contusion after bumper car collision: case report and review of the literature. Respir Med Case Rep.

[22] Weigeldt M, Paul M, Schulz-Drost S (2018). Anesthesia, ventilation and pain treatment in thoracic trauma. Unfallchirurg..

[23] Yuehua X, Ping J (2015). Multifactorial study of pulmonary insufficiency in patients with severe multiple injuries. Modern Pract Med.

